# Editorial: Capturing Biological Complexity and Heterogeneity Using Multidimensional MRI

**DOI:** 10.3389/fphy.2022.950928

**Published:** 2022-07-04

**Authors:** Mustapha Bouhrara, Jana Hutter, Dan Benjamini

**Affiliations:** 1 Magnetic Resonance Physics of Aging and Dementia Unit, National Institute on Aging, National Institutes of Health, Baltimore, MD, United States,; 2 Centre for the Developing Brain, King’s College London, London, United Kingdom,; 3 Biomedical Engineering Department, King’s College London, London, United Kingdom,; 4 Multiscale Imaging and Integrative Biophysics Unit, National Institute on Aging, National Institutes of Health, Baltimore, MD, United States

**Keywords:** multidimensional, MRI, diffusion, relaxation, medical imaging, microstructure, exchange

In the context of medical imaging, the term “resolution” most often refers to the measure of the smallest object that can be resolved from the image, i.e., *spatial* resolution. The two most prominent modalities that offer comparable spatial resolution on the order of 1 mm^3^ in clinical applications, computed tomography (CT) and magnetic resonance imaging (MRI), provide the ability to noninvasively probe biological tissue in depth and *in vivo*. While important macroscopic information resides at the 1 mm length scale, such as white and gray matter discrimination or visible lesions identification, some tissue level and definitely cell-level processes remain elusive.

Despite its limited spatial resolution, MRI and more precisely nuclear magnetic resonance (NMR) is a spectroscopic modality, and therefore its *spectral* resolution is a second, more unique type of resolution that is leveraged in MRI applications. The idea to jointly encode more than a single MR parameter (e.g., relaxation, diffusion), or so-called dimension, was first proposed more than 4 decades ago [1], and was repeatedly shown to enhance the spectral resolution in a range of complex systems, from biological tissue [2, 3] to sedimentary rocks [4]. Spectral resolution in the current context should not be confused with the more common use of the term in Fourier transform NMR spectroscopy with multidimensional correlation of, for instance, 1H and 15N chemical shifts. In biological applications, relaxation-diffusion multidimensional MR provides information from compositionally- and microstructurally-diverse environments, thus offering a probe of tissue heterogeneity. More recently, the use of multidimensional MRI to capture sub-voxel information has been gaining attention, leading to increased specificity and sensitivity towards a range of biological states and diseases [5–9].

The goal of this Research Topic was to give a platform to introduce novel and cutting-edge methods that utilize MRI as a multidimensional tool, and to advance the field towards more refined, sensitive, and specific imaging. The papers here focused on taking advantage of untapped dimensions to increase the spectral resolution, and as a result, to increase the effective spatial resolution by mapping sub-voxel components that describe physical and chemical properties of the different microenvironments in complex and heterogeneous biological materials.

The first few papers in this Research Topic are focused on introducing new frameworks to process multidimensional diffusion data. Magdoom et al. explore a novel diffusion tensor distribution (DTD) paradigm exploiting its ability to probe features below MRI voxel sizes to study neural tissue. Results in *ex vivo* spinal cord tissue of macaque monkeys showed its ability to detect the boundaries of cortical layers in the higher moments of the mean diffusivity (MD) spectrum consistent with histology, revealed crossing and splaying fibres penetrating into the cortex, and skewed fiber diameter distributions in the white matter tracts. Different diffusion encoding properties span a new multidimensional parameter space allowing to detect hidden tissue properties. Song et al. present a new approach to estimate the full DTD (FDTD) without any constraints on the functional form of the distribution and/or the symmetries of the tensor components, based on data acquired with multi-shell single diffusion encoding data up to very high diffusion weighting (300 mT/m). They show their approach for data acquired on the Connectom scanner for one volunteer and two patients with diffuse gliomas. They introduce the use of a k-means algorithm to cluster the FDTD data, and demonstrate the potential utility for characterization of tissue types in gliomas. By incorporating a more realistic diffusion signal model, Boito et al. introduce the confinement tensor distribution (CTD) as an augmented approach of the previously suggested DTD for analyzing restricted and anisotropic diffusion in biological tissues. In their work, they consider a model that takes restriction into account, thus replacing the multi-exponential signal representation inherent to DTD. They introduce expanded theory, extensive numerical simulations, and then apply the CTD framework on a human brain dataset, providing new insights regarding the structural composition of complex media. Shifting the focus to exchange, Cai et al. propose an acquisition scheme to independently characterize restriction and exchange with various diffusion exchange spectroscopy (DEXSY) measurements. Results in a viable *ex vivo* mouse spinal cord tissue using a low-field static NMR system demonstrate the newly disentangled effects of restriction and exchange. Combining multiple diffusion encoding paradigms with exchange mechanisms opens a different multidimensional space, allowing to separate normally confounded properties.

The two next papers incorporate relaxation to the diffusion encoding scheme and introduce new processing pipelines. Narvaez et al. join together diffusion-relaxation correlation, time/frequency-dependent diffusion, and tensor-valued encoding into a common data acquisition and analysis framework. They leverage the inherent frequency dependence of the diffusion gradients waveforms to resolve a “massively multidimensional” diffusion-relaxation correlation dataset in *ex vivo* rat brain, with subsequent histological analyses and comparisons, and demonstrate unprecedented level of detail within the brain. Using a different approach and pulse sequence, Benjamini et al. propose applying an adaptive nonlocal multispectral filter denoising procedure to increase the effective signal-to-noise ratio of multidimensional MRI data, achieving significant reduction in data requirement and subsequent acquisition acceleration. They demonstrate this approach by reprocessing data from a recent study that identified imaging biomarkers of subtle axonal injury pathology in *ex vivo* human Corpus Callosum samples using diffusion-relaxation MRI. The obtained results show improved accuracy of the reconstructed injury MRI biomarker maps, while reducing the required data amount.

With a departure from the central nervous system, Lasič et al. present a novel prediction framework for time-dependent diffusion effects using b-tensor encoding. Experimental data from *ex vivo* mouse and pig hearts and the resulting strong and predictable variation of MD time-dependent diffusion paves the way for future *in vivo* cardiac experiments. This is a great illustration of how multidimensional MRI–here different shapes of the b-tensor in combination with time-dependence–captures otherwise not accessible properties of biological tissue. Lastly, Afzali et al. provide a review focusing on applications of tensor valued encoding, a prominent diffusion MRI technique, in the brain and in several organs in the body including the heart, breast, kidney and prostate, to infer tissue microstructural information that is not accessible using traditional diffusion MRI techniques. The application of this technique to various diseases and conditions is described. The review also provides a brief description of the different diffusion encoding schemes and technical considerations, with an exhaustive list of corresponding literature.

In conclusion, this Research Topic illustrates the incredible versatility and untapped potential of MR in gleaning information from heterogeneous biological systems. While it is becoming increasingly clear that no single MR contrast mechanism is able to grasp the full complexity of our biology, the concept of jointly encoding several MR contrasts and thus shedding light on different tissue properties simultaneously is especially enticing. We hope that the Research Topic of works will serve as a catalyst for more wide-spread use of multidimensional MRI.
